# Alcohol environment, gender and nonfatal injuries in young people. An ecological study of fourteen Swedish municipalities (2000–2005)

**DOI:** 10.1186/1747-597X-7-36

**Published:** 2012-08-21

**Authors:** Richard A Dale, Marie Hasselberg, Max Petzold, Gunnel Hensing

**Affiliations:** 1 Department of Social Medicine, The Sahlgrenska Academy at the University of Gothenburg, PO Box 453, SE-405 30, Gothenburg, Sweden; 2 Department of Public Health Sciences, Division of Global Health/IHCAR, Karolinska Institute, Stockholm, Sweden; 3 Centre for Applied Biostatistics, The Sahlgrenska Academy at the University of Gothenburg, Gothenburg, Sweden

**Keywords:** Children, Young adults, Alcohol access, Per capita alcohol consumption, Crimes against alcohol laws, Nonfatal injuries, Sweden, Municipality level, Gender

## Abstract

**Background:**

Sweden has had a restrictive alcohol policy, but there are gender and geographical differences in alcohol consumption and injury rates within the country. Whether and how the Swedish alcohol environment influences gender differences in injuries in young people is still unclear. Thus, the aim of this study was to analyse the associations between the local alcohol environment and age- and gender-specific nonfatal injury rates in people up to 24 years in Sweden.

**Methods:**

The local alcohol environment from 14 municipalities was studied using indicators of alcohol access, alcohol consumption and alcohol-related crimes. A comprehensive health care register of nonfatal injuries was used to estimate mean annual rates of nonfatal injuries by gender and age group (2000–2005). Pearson’s correlation coefficients were used to analyse linear associations.

**Results:**

Associations were shown for both alcohol access and alcohol consumption with injury rates in boys aged 13–17 years; no other associations were observed between alcohol access or per capita alcohol consumption and nonfatal childhood injuries. The prevalence of crimes against alcohol laws was associated with injury rates in children of both genders aged 6–17 years.

**Conclusions:**

This study found no strong area-level associations between alcohol and age and gender specific nonfatal injuries in young people. Further, the strength of the area-level associations varied by age, gender and type of indicator used to study the local alcohol environment.

## Background

Alcohol is a recognized risk factor for different types of injuries, both unintentional (e.g. falls) and intentional (violence and suicide) [1], and injuries are the main cause of death and disability among young people in European countries [2]. Studies have found that high local alcohol outlet density [3-5], high per capita alcohol consumption [6-8] and detrimental societal drinking patterns [9] are associated with fatal and severe injuries in the adult population. Stockwell et al. [10] estimated that an increase of the private store density was associated with an increase in rates of alcohol-related death. Studies of college students have found that easy access to alcohol at the neighbourhood level is associated with severe and fatal injuries in young adults (18–24 years) [11,12]. Additionally, studies from the US and Australia have shown that easy local alcohol access is associated with adolescent alcohol consumption and alcohol abuse [13-15]. However, knowledge about the associations between easy alcohol access and children’s injuries is scarce. It seems important to fill this gap given the growing evidence showing that local characteristics of the socio-cultural structures shape opportunities, preferences, behaviours and health outcomes [16,17].

No Scandinavian studies have explored before the association between local alcohol environment and nonfatal childhood injuries. Two studies from California, however, found that easy purchase of alcohol was associated with violence-related injuries in children [18,19]. Two other studies have shown an association between rates of driving while intoxicated with alcohol (DWI) and fatal traffic injuries in children who are car passengers, pedestrians and cyclists [20]. Those studies suggest that alcohol-related problems are not limited to alcoholics. These studies also show that fatal and intentional childhood injuries are influenced by adults’ alcohol behaviors and their access to purchase alcohol. Potential mechanisms behind the relationships between alcohol availability and injuries are complex and theoretical models are scarce [4]. A simplified explanation is that greater availability is related to greater use and increased risk for alcohol-involved injuries. Studies have shown that also low levels of alcohol consumption contribute to higher risk of injury and that this risk increases with increasing levels of consumption [21]. Alcohol consumption by parents and other caretakers while supervising children also increase the risk for both intentional and unintentional injuries among children. Apart from these explanations, access to alcohol may also interact with other contextual factors that could increase the risk of injuries. A study from the US has indicated that areas with many bars, high alcohol consumption and complex roadway systems could increase the risk of pedestrian injuries [22].

Sweden has had one of the most restrictive alcohol policies in the Western world with a state-owned alcohol retailing monopoly, high alcohol taxes and a very strict regulation for DWI [23,24]. Buying alcohol in restaurants or bars is legal from the age of 18 and at the alcohol retailing monopoly from the age of 20. Nevertheless, alcohol accounted for 25% of deaths in people up to 50 years old, and about 10% of person-years of life lost in Sweden from 1992 to 1996 [25]. Sweden’s membership in the European Union (1995) contributed to a more liberal alcohol policy, easier access to alcohol and higher per capita alcohol consumption than prior to the entrance to the European Union [23]. The average per capita alcohol consumption in Sweden increased from eight litres in 1996 to ten litres in 2002 [26]. The Swedish alcohol-related injury rates (per 10,000 inhabitants) increased from 61 to 80 between the years 1996 and 2005 [27]. Whether and how this new alcohol environment in Sweden influences the risk of injuries among young people is still unclear. Nonfatal injuries in children and youth are still common in Sweden, in spite of advanced safety regulations and policies. Nonfatal childhood injuries contribute to high cost for health services, society, and individuals, including long-term consequences for young people [2]. New perspectives are needed in research on injury prevention and safety promotion, and few studies have focused on the local alcohol environment and its associations with injuries, not only in those consuming alcohol, but also in other individuals. It is well known that boys, and in particularly young men, have more access to alcohol, consume more alcohol and are over-represented in registered injuries [15,28-30]. This study aimed to fill this knowledge gap and to provide new insights for safety promotion and injury prevention. Therefore, the aim of this study was to analyse the associations between the local alcohol environment and age- and gender-specific nonfatal injury rates in people up to 24 years in Sweden.

## Methods

This ecological study comprised 14 municipalities in the southwest of Sweden, in an agricultural and manufacturing area, with 40% arable land compared with 8% for Sweden as a whole. The municipality-level was selected since it is the smallest spatial, administrative and political unit with a significant degree of autonomy, and it is responsible for the health of its population, which includes legislation and enforcement of local alcohol policies. The average annual population in the included municipalities from 2000 to 2005 ranged from 6,000 to 50,000 inhabitants (N = 256,000 inhabitants). Thirty percent of the inhabitants were 24 years of age or under (n = 74,500 inhabitants). This region was selected because it has a long and unique record of nonfatal injuries that will be described in more detail below.

### Alcohol data

**Figure 1:**
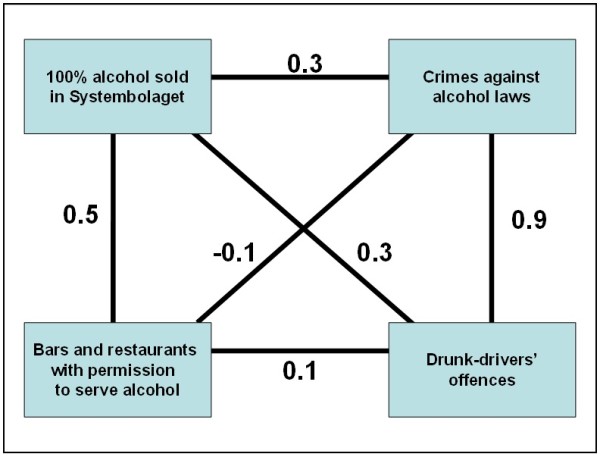
Pearson’s correlations coefficient between the four alcohol indicators.

“Alcohol environment” was defined in this study as the dominant societal attitudes and practices in terms of alcohol consumption and related behaviours. Three Swedish databases were scrutinised to obtain municipal indicators that could characterise the local alcohol environment: Statistics Sweden (http://www.scb.se), the Swedish National Institute of Public Health (http://www.fhi.se), and the Swedish National Council for Crime Prevention (http://www.bra.se). Four indicators were selected to characterise three aspects of the local alcohol environment: alcohol access, alcohol consumption, and alcohol-related crimes (see Table 1). Figure 1 shows the strength and direction of the correlations between the four indicators of the alcohol environment.

**Table 1:** Contextual background of the 14 municipalities in comparison with Sweden

**Socio-demographic background**	Mean (SD)^*^	Range^**^	**Sweden**
Annual total population (per 1,000 inhabitants)	18 (13)	5 – 49	8800
Population of young people (0–24 years) (%)	33 (1)	27 – 31	31
Population living in the urban area (%)	68 (12)	42 – 85	80
Children living in a household with social welfare (%)	6 (2)	3 – 12	18
**Alcohol context**			
Density of restaurants and bars licensed to serve alcohol per 10,000 inhabitants^**§**^ (local alcohol access)	13 (5)	8 – 26	14
Litres of 100% alcohol sold per 10,000 inhabitants 15 years and older^**§**^ (per capita alcohol consumption)	4 (1)	3 – 5	5
Driving under the influence of alcohol (per 10,000 inhabitants)	14 (3)	9 – 18	17
Registered crimes against alcohol laws^¥^ (per 10,000 inhabitants).	17 (4)	11 – 26	4
Alcohol intoxication, women 15-24y (per 10,000)	18 (6)	10 – 28	19
Alcohol intoxication, men 15-24y (per 10,000)	22 (7)	10 – 36	20
**Injury context**			
Mean annual fatal injury rate among females (per 10,000)	4 (1)	2 – 6	3
Mean annual fatal injury rate among males (per 10,000)	7 (2)	4 – 10	7

^*^ Mean is the annual mean from the 14 municipalities and from the years under study (2000–2005). (SD) is the standard deviation from the mean.

^**^ Range is the description of the lowest and the highest mean value from the years 2000 to 2005 between the municipalities under study.

^§^ Alcohol sold in the Swedish state-owned alcohol retail monopoly.

^¥^ Crimes against alcohol laws related to production, sale and consumption of illegal alcohol.

Density of restaurants and bars licensed to serve alcohol per 10,000 inhabitants was used to measure “local alcohol access” [31]. Litres of 100% alcohol sold by the alcohol retail monopoly per 10,000 inhabitants aged fifteen or older were used to measure “per capita alcohol consumption” which is the common way to describe this measure based in official statistics from the Swedish retail monopoly [6-8]. The measure pools all alcohol sales and converts to 100% pure alcohol. “Alcohol-related crimes” were measured by two indicators: annual rates of driving while intoxicated with alcohol per 10,000 inhabitants [24] and annual rates of crimes against the Swedish alcohol laws (related to production, sale and consumption of illegal alcohol) per 10,000 inhabitants. According to the Swedish laws, the production of alcohol can only be made by licensed producers. A common crime is private small scale productions at home. Further, the alcohol law restricts sales of alcohol which mainly can be done by specific shops (Systembolaget) owned by the State or at restaurants. A common offence against this part of the law is sales of illegal import from countries with lower alcohol taxes such as Denmark and Germany. Finally, consumption of alcohol in public places is restricted and offences against this are rather common crimes. It is important to mention that the studied region had rates of crimes against alcohol laws (17/10 000 inhabitants) four times higher than Sweden as a whole (4/10 000 inhabitants). Each selected indicator was registered annually for each municipality during the study period (2000–2005). Unfortunately, specific information about alcohol consumption among youths was not available at the municipality level. The only local information on youth intoxication by alcohol was recorded in close association with suicide attempts and so was not included in the analysis. Consequently, we could not control for the potential confounding effect of young people’s alcohol consumption. No linear association was observed between any of the studied alcohol variables and level of poverty or level of urbanization (analysis not presented here).

### Injury data

The annual number of injuries per municipality (2000–2005) was obtained from the Skaraborg Local Injury Surveillance System (SLISS). The database covered four local public hospitals and 25 primary healthcare centres (PHC). Each municipality had at least one PHC. The injury information was systematically collected with two instruments: the clinical record completed by the treating physician and a self-administered questionnaire collecting demographic data and information about the event (when, where, and how the injury took place, and during which activity). Injuries were coded according to the classification system of the European Home and Leisure Accident Surveillance System and the ICD-10 (chapter XX). However, the data did not allow us to distinguish between intentional and unintentional injuries. Each new registered injury episode was treated as a single case. There were very few repeated cases but those who occurred were treated as a new case. Independently of where the injury occurred or where the injury was treated the case was registered according to the area of residence in this at the home municipality of the injured person. Cases coded “no injury found” at clinical evaluation and dental fractures were excluded. Injury database management and analyses used anonymous information (without the patient’s personal identification number) in accordance with the Swedish legislation for registers. There is no information on alcohol consumption in the SLISS.

The injury data were stratified by gender and four age groups (0–5 years, 6–12 years, 13–17 years, and 18–24 years). These age limits were chosen to include both children and youth in accordance with the United Nations’ definitions. This was done since earlier studies have shown that alcohol consumption [15] and alcohol-related problems [1,28,29], and childhood injuries are more common in boys and the gender gap increases with the age [30]. Furthermore, we assumed age-related differences in development, social practices, and exposure to risk of injury. Annual gender- and age-specific injury rates were computed using the gender- and age-specific averages for the populations in each municipality. Data were retrieved from Statistics Sweden. Since we were dealing with small municipalities with relatively low occurrences of injuries, the annual rates were transformed into mean annual rates (2000–2005) to obtain more stable values for the analyses.

### Statistical analysis

Pearson’s correlation coefficient (SPSS version 15.0) was selected to analyse the strength and direction of linear associations. A correlation close to zero means that there is no linear relationship, whereas a correlation close to one or to minus one indicates a strong linear relationship between the studied variables. The analyses in this study were based on a small sample (n = 14) and therefore the level of significance will not be acknowledged. Instead, the following sections will discuss linear correlations of −0.4 to 0.4 and stronger (r ≤ −0.4 or ≥ 0.4). The chosen cut off limits are arbitrary but reasonable in terms of clarity in linear pattern*,* and perceived as at least a moderate linear correlation*.* The strength and direction of the gender- and age- specific correlations were compared to determine whether the local alcohol environment was associated with gender differences in nonfatal injuries.

## Results

Forty-four per cent (n = 11,020) of the annual average number of registered injuries over the 14 municipalities during the years 2000 to 2005 were among people aged 24 years or under. Rates of nonfatal injuries were higher among males than among females of all ages (see Table 2). Young adults (18–24 years) had the largest gender difference: males had nonfatal injury rates twice as high as those of females.

**Table 2:** Mean annual nonfatal injury rates by sex and age and age specific sex ratios in the 14 municipalities (2000–2005) with their specific 95% confidence interval

**Age**	**Nonfatal injuries/1000 inhabitants**					
	**Girls**		**Boys**		Sex ratio^*^	
	**Mean**	**95% CI**	**Mean**	**95% CI**	**Mean**	**95% CI**
0-5 years	131	(119 – 144)	158	(147 – 169)	1.2	(1.2 – 1.3)
6-12 years	139	(131 – 148)	181	(170 – 192)	1.3	(1.2 – 1.4)
13-17 years	119	(110 – 129)	182	(170 – 194)	1.5	(1.5 – 1.6)
18-24 years	89	(83 – 95)	181	(169 – 194)	2.1	(1.9 – 2.2)

^*^ Mean sex ratio (male rate/female rate).

### Alcohol environment and nonfatal injuries

**Figure 2:**
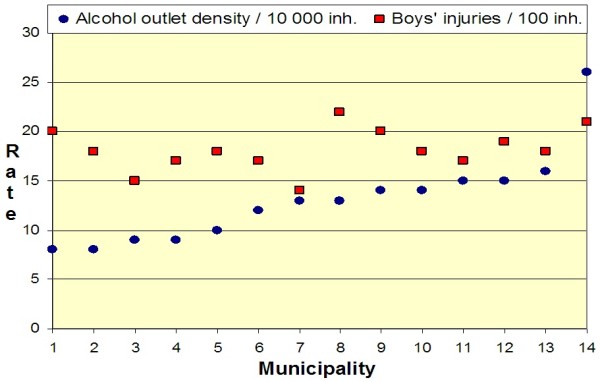
**Mean annual distribution of alcohol outlets and nonfatal injuries in boys aged 13–17 years for each of the studied 14 municipalities (2000–2005).** Municipalities are ranked by level of alcohol outlet density.

**Figure 3:**
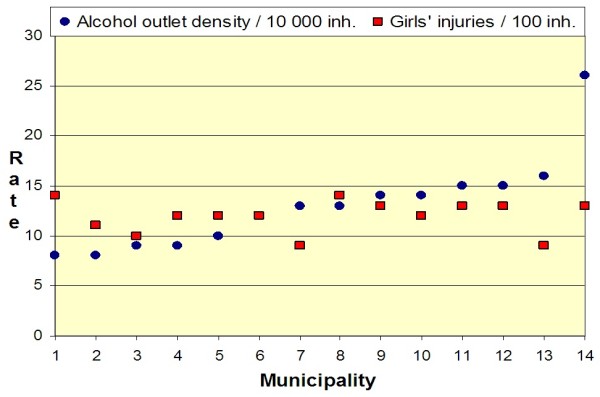
**Mean annual distribution of alcohol outlets and nonfatal injuries in girls aged 13–17 years for each of the studied 14 municipalities (2000–2005).** Municipalities are ranked by level of alcohol outlet density.

Municipalities with high rates of crimes against alcohol laws had high injury rates in children aged 6–17 years. Positive correlations (r ≥ 0.5) were observed between crimes against alcohol laws and nonfatal injuries in both boys and girls aged 6–12 and 13–17 years (see Table 2). The prevalence of driving while intoxicated with alcohol also showed a positive correlation (r = 0.4) with nonfatal injuries in boys and girls aged 13–17 years. Local alcohol access and per capita alcohol consumption were positively correlated (r = 0.4) with nonfatal injuries in boys 13–17 years (see Figure 2) but not with injuries in girls at any age (see Figure 3). No strong correlation was observed between any of the studied indicators of alcohol environment and nonfatal injuries in children of either gender aged 5 years or under or in young adults (18–24 years) of either gender.

### Gender specific analyses of the association between alcohol and nonfatal injuries

The gender specific analyses of nonfatal injuries (see Table 3) showed that local alcohol access and per capita alcohol consumption were positively correlated (r = 0.4) with nonfatal injuries in boys aged 13–17 years, but there was no such correlation for girls in that age group (r = ± 0.1). No gender discrepancies were found in the direction and strength of the correlations between any of the studied indicators of alcohol environment and nonfatal injuries in children aged 5 years and under and in young adults (18–24 years).

**Table 3:** Pearson’s correlation values based on the 14 municipalities, by indicators of local alcohol environment, nonfatal injuries, age, and sex

**NONFATAL INJURIES**^§^	**0-5 years correlations**		**6-12 years correlations**		**13-17 years correlations**		**18-24 years correlations**	
	**Girls**	**Boys**	**Girls**	**Boys**	**Girls**	**Boys**	**Girls**	**Boys**
Local alcohol access	0.0	0.3	−0.2	0.2	0.1	0.4	0.2	0.2
Per capita alcohol consumption	0.0	0.2	−0.2	0.2	−0.1	0.4	0.2	0.2
Driving while intoxicated by alcohol	−0.1	0.1	0.3	0.3	0.4	0.4	0.3	0.3
Crimes against alcohol laws	0.2	0.2	0.6^*^	0.5	0.6^*^	0.6^*^	0.3	0.3

^§^ = All registered episodes of nonfatal injuries, excluding cases coded as “no-injury found”.

Note: Correlation “0” = no linear association, and correlation “1” or “-1” = strong linear positive or negative association.

^*^P-value < 0.05.

## Discussion

This is the first study in Sweden analysing ecological associations between the local alcohol environment and non-fatal injuries in children and young people. Except for the associations between alcohol access and alcohol consumption with injury rates in boys aged 13–17 years, no associations were observed between alcohol access or per capita alcohol consumption and nonfatal childhood injuries. Municipalities with high rates of crimes against alcohol laws, however, had high injury rates in both boys and girls aged 6–17 years. High rates of driving while intoxicated with alcohol were associated with non-fatal injuries in boys and girls aged 13–17 years. No association was found between any of the indicators of alcohol environment and nonfatal injuries in children aged 5 years or under or in young adults (18–24 years).

Since many studies have shown that alcohol consumption and alcohol-related problems have increased in the adult Swedish populations [23,24,27], positive associations were expected between the alcohol environment and injuries in young people in this study. However, except for the results obtained in boys aged 13–17 years no other association at the municipality-level was found between local alcohol access or per capita alcohol consumption and nonfatal childhood injuries. It has been suggested elsewhere that modifications in the local alcohol environment lead to individual changes in drinking behavior and in alcohol-related problems [32]. Thus, one possible explanation for the lack of area-level associations between alcohol access or per capita alcohol consumption and nonfatal childhood injuries could be the restrictive alcohol policies in Sweden. Apart from policies and restrictions, local drinking patterns might also be influenced by local norms and social control in some of the smaller municipalities with high participation rates in sport clubs, social associations and churches. Unfortunately, variations in this respect between the municipalities were not possible to study due to the small number (n = 14) of municipalities involved in injury registrations with SLISS. Since this is the first time that the local alcohol access and per capita alcohol consumption is studied in Sweden in association to nonfatal childhood injuries, another explanation may be that these alcohol indicators and nonfatal childhood injuries are simply not associated in Sweden at all, or at least not in smaller semi-rural municipalities as those studied here.

In congruence with earlier studies [7,8] we found a positive area-level association between per capita alcohol consumption and injuries in boys 13–17 years. One important difference between this ecological study and the earlier studies [7,8] is that they used fatal injuries at national level, whereas this study used nonfatal injuries at the municipality-level. Another important difference is that earlier studies used populations aged 15 and over. Since there are studies suggesting that adolescent drinking patterns mirror those of adults [33] it is possible that adolescent alcohol consumption is an important determinant of the observed association. There were, however, no data available to control for that possibility.

The positive area-level associations in boys and the negative associations in girls between alcohol access, alcohol consumption, and injuries in children aged 6–17 years could have at least three explanations. One explanation, not explored here, is that boys may have higher access to alcohol [15] and consume more alcohol than girls even if the reported differences are higher in older age groups than in younger [15,28,29]. It is also possible that local gender norms and opportunities may influence not only access to and consumption of alcohol but also the access to and choice of leisure time activities [34]. Boys, for example, may have had higher exposure to injury risks than girls due to higher access to sports arenas and higher mobility, but we were not able to control for this in the analysis. Another explanation is that boys may have higher outdoor injuries and intentional injuries than girls [29], which could also explain the observed gender differences in strengths and direction of the associations between alcohol access and per capita alcohol consumption and injuries in children aged 13–17 years. A reason for not including possible confounders in the analysis is twofold. First, even if Sweden has rich public statistics there is a lack of specific data at the municipality level. National statistics are more available but given the large variation in municipality characteristics in Sweden the national mean numbers cannot reflect local circumstances. Sweden is a vast country but the population density is low and local variations and high self-determinations in municipalities are high. Thus, a lack of relevant statistical information on possible confounders at the municipality level limited our confounding analysis. Second, we were limited also by the number of municipalities participating in the injury registration. The data is descriptive and constitutes a basis for generating hypotheses to be explored in future studies.

The two ecological indicators of local alcohol-related crimes (DWI and crimes against alcohol laws) showed positive area-level associations with the occurrence of nonfatal injuries in both boys and girls aged 13–17 years. No earlier study was found exploring these ecological indicators in association with childhood injuries, it is important to discuss these results in relation to the validity of the alcohol indicators. The Swedish legislation has severe penalties for DWI offences [24], and Hubicka, Bergman and Laurell [35] have suggested that the registration of DWI offences is strongly influenced by active control by the police. Since the studied municipalities are mainly rural, it is highly probable that the registered DWI offences in the studied region were associated with damage to a property or to violent injury. Hubicka and Bergman [35] also found an association between DWI and criminality and antisocial behaviours. Therefore, it is possible that the two ecological indicators of alcohol-related crimes are more closely related to the level of overall criminality than merely to the alcohol environment. Additionally, since mean annual rates for 2000–2005 were used to stabilize the data, low numbers of alcohol-related crimes might have produced some random difference in our data compared with Sweden as a whole. Finally, because the 14 municipalities share the same Police Administration and the same County Administrative Board, we do not assume large geographical differences in enforcement, policies or practices between the studied municipalities.

In this study none of the ecological indicators of the local alcohol environment was associated with the occurrence of nonfatal injuries in young adults (18–24 years). This result was unexpected since previous area-level studies [11,12] have found a positive association between different indicators of alcohol use, alcohol behaviour and injuries in young adults. A possible explanation for the absence of area-level associations in this age group might be methodological and related to health care seeking behaviour for minor injuries compared to younger age groups. Young adults have the highest alcohol consumption of all age groups in Sweden and it is possible that they avoid seeking health care for minor injuries occurring while they are under intoxication. Self care might also be more common in this age group compared to the younger ones. Another possible explanation is that better coping strategies for avoiding injuries in hazardous situations have been developed with age resulting in lower injury prevalence rates.

It is well known that alcohol consumption and alcohol-related crimes are unevenly distributed between women and men. However, few studies have analysed gender in relation to risk exposure measured at the municipality level. The observed gender differences in the area-level associations between per capita alcohol consumption and nonfatal injuries in children aged 6–17 years show that gender is an essential determinant of social outcomes, including injuries, and further studies should take this factor into account. It is also important that statistics and other sources of information present data for both genders. Boys, and in particular young men, are not only consuming more alcohol and more often involved in alcohol-related crimes they also have a higher burden of injuries. This study suggests that the local alcohol environment might be an important factor to study further in the future. It would be of specific interest to be able to better separate individual-level exposure from the municipality-level of exposure and also to identify possible interactive effects. Another perspective is important to mention. The area-level association between crimes against alcohol laws and injuries was similar between girls and boys. In the injury register it is not possible to separate violent related injuries otherwise, it would have been interesting to separately analyse the association between crimes against alcohol laws and injuries in girls and boys contributing to the understanding of the influence of alcohol and violence against girls and young women. The underreporting of violence behind injuries is expected to be higher among girls and young women due to the stigma and shame associated with violence exposure in girls and women.

## Methodological considerations

As far as we know, this is the first European study exploring age- and gender-specific associations between alcohol environment and nonfatal childhood injuries at the municipality level. The injury database allowed us to use a wide spectrum of nonfatal injuries, with data collected from both hospital and primary healthcare clinics. The number of registered injuries in the study group was 77,000 (2000–2005) and represents almost all nonfatal injuries for which treatment was sought in this region. The study was limited to 14 small neighbouring municipalities due to the coverage of the injury register, which reduced the statistical power. The municipality level of the study means that the findings cannot be interpreted as family, neighbourhood, or individual associations between alcohol and nonfatal injuries. However, the municipality level is relevant because of the administrative and political importance of municipalities in Sweden. Measures for alcohol harm reduction, other than legislation, taxation, and the alcohol retail monopoly, are usually based on the municipality level in Sweden, and these data thus have relevance for public health practice. From the public health science perspective, it has been suggested that we need more knowledge of how characteristics at levels above the personal, familial, and social influence individual health [36,37]. This study is a contribution to such research.

## Conclusions

In spite of the observed differences in injury rates by age groups and gender this study found no strong area-level association between alcohol and age and gender specific nonfatal injuries in young people. Further, the strength of the area-level associations varied by age, gender and type of indicator used to study the local alcohol environment. This result suggest the importance of specific age and gender analysis for better understanding of potential contributors to injury risk for different age groups and genders. More knowledge is needed to understand whether the restrictive alcohol policies in Sweden still play an important role in the prevention of childhood injuries.

## Competing interests

The authors declare that they have no competing interests.

## Authors’ contributions

All authors participated in its design and helped to draft the manuscript. RAD carried out all the analyses, assisted by MP. All authors read and approved the final manuscript.
